# Block Copolymer-Assisted Synthesis of Iron Oxide Nanoparticles for Effective Removal of Congo Red

**DOI:** 10.3390/molecules28041914

**Published:** 2023-02-17

**Authors:** Mohan K. Bhattarai, Moses D. Ashie, Sita Dugu, Kiran Subedi, Bishnu P. Bastakoti, Gerardo Morell, Ram S. Katiyar

**Affiliations:** 1 Department of Physics, University of Puerto Rico, P.O. Box 70377, San Juan, PR 00936-8377, USA; 2 Department of Chemistry, North Carolina A&T State University, 1601 East Market Street, Greensboro, NC 27411, USA; 3 Analytical Services Laboratory, College of Agriculture and Environmental Sciences, North Carolina A&T State University, 1601 East Market Street, Greensboro, NC 27411, USA

**Keywords:** Rietveld, vibrational study, IONPs, block copolymer, hydrothermal, adsorption, α-Fe_2_O_3_, superparamagnetism

## Abstract

Iron oxide nanoparticles (IONPs) were synthesized via a block copolymer-assisted hydrothermal method and the phase purity and the crystal structure were investigated by X-ray diffraction. The Rietveld analysis of X-ray diffractometer spectra shows the hexagonal phase symmetry of α-Fe_2_O_3_. Further, the vibrational study suggests Raman active modes: 2A1g + 5Eg associated with α-Fe_2_O_3_, which corroborates the Rietveld analysis and orbital analysis of 2PFe. The superparamagnetic behavior is confirmed by magnetic measurements performed by the physical properties measurement system. The systematic study of the Congo red (CR) interaction with IONPs using a UV-visible spectrophotometer and a liquid chromatography–tandem mass spectrometry system equipped with a triple quadrupole mass analyzer and an electrospray ionization interface shows effective adsorption. In visible light, the Fe_2_O_3_ nanoparticles get easily excited and generate electrons and holes. The photogenerated electrons reduce the Fe^3+^ ions to Fe^2+^ ions. The Fe^2+^/H_2_O_2_ oxidizes CR by the Fenton mechanism. The strong adsorption ability of prepared nanoparticles towards dyes attributes the potential candidates for wastewater treatment and other catalytic applications.

## 1. Introduction

Metal oxide nanoparticles show size-dependent physical, chemical, electronic, and magnetic properties that constitute versatile platforms for producing materials with possible applications in catalysis and magnetic and optical devices [1,2,3]. The studies have shown that the catalytic and magnetic properties of nanoparticles can be quite different from other bulk materials. When the size of the nanoparticles is below a critical value, or depending on the material, nanoparticles (NPs) behave like a giant paramagnetic atom with a single magnetic domain, exhibiting superparamagnetic behavior [2]. Fe_2_O_3_ nanoparticles show superparamagnetic with a size-dependent blocking temperature [4] and enhanced catalytic properties compared to bulk materials [5]. Since the surface-to-volume ratio increases with decreasing nanoparticle diameters, surface effects contribute to those properties. However, there are some challenges in tuning the magnetization, stability, capture efficiency, and surface properties of iron oxide nanoparticles (IONPs) [2]. The nanomaterials with a high surface area are important in established processes such as catalysis and molecular separations alongside emerging technologies for energy and health [6,7]. Multiple components, such as the size of the iron oxide crystals, the charge, the nature of the coating, and the hydrodynamic size of the coated particle, have to be measured accurately, as far as possible, for their effective application [8].

Improvements to metal oxide nanoparticle characteristics, such as enhanced magnetic properties, non-biofouling surface coatings, and the integration of multifunctional ligands, continue improving its potential applications. Superparamagnetic iron oxide nanoparticles (SPIONs) are among the most used nanoparticle in biomedical fields as contrast agents for magnetic resonance imaging, drug delivery systems, or hyperthermia agents in cancer therapies [9]. The superparamagnetic and high surface area of Fe_2_O_3_ nanomaterials can remove heavy metal arsenic ions in water, promising materials for wastewater treatment [10] and different types of coupling reactions [6]. The magnetic properties of iron oxide nanoparticles make them easily recyclable and can be used multiple times. This facilitates the application and cost management in the use of magnetic nanoparticles, while at the same time, minimizing the number of times materials have to be synthesized. The Fe_2_O_3_ nanoparticles were successfully used for the catalytic destruction of ethane, where the presence of oxygen vacancies and lattice defects enables the oxygen transfer speed and redox capabilities of Fe_2_O_3_ [11].

Water purification has recently gained significant attention as it is contaminated due to different anthropogenic activities. Pesticides from agricultural activities, fecal-related contaminants releasing pathogens, domestic wastes, and industrial effluents are a few sources of water pollutants. Among the chemical pollutants, organic dyes, due to their toxic nature, are one of the major contaminants of industrial water. Therefore, continuous efforts are ongoing to effectively deal with industrial wastewater containing organic pollutants. Congo red (CR) is widely used to dye fabrics and stain tissues. Despite its usefulness, releasing large quantities of CR can be harmful and cause environmental pollution. Carcinogenicity and various toxicities associated with CR that affect flora, fauna, and humans mean there is an urgency for removing CR from wastewater.

Nanotechnology has been an exceptional development for water treatment compared to costly conventional treatment methods and remediation from microorganisms and organic dyes [12]. Catalytic adsorption of dyes is, therefore, of great interest as the molecules of the dyes are removed for safe industrial handling of excess pollutants and safe disposal of effluents. The need for remediation of wastewater challenges has caused the development of different heterogeneous inorganic catalysts/adsorbents, which find application in adsorption and degradation [13,14]. IONPs are abundant, easily accessible, environmentally compatible, and have high stability, low cost, and suitable magnetic properties. Various methods have been developed to prepare IONPs [15,16,17,18], although they are prone to aggregate during synthesis, which limits their applications. Many physical and chemical approaches are used to avoid this limitation. Numerous synthetic routes have been developed to produce magnetic nanoparticles with good control of shape, size, and distribution. Iron oxide, in most cases, can be synthesized either by top-down or bottom-up techniques. Lots of different mechanical and physical techniques, such as ultrasonication, irradiation, laser ablation, microwave, and electrochemical, and physical vapor deposition, are used in top-down approaches to produce magnetic nanoparticles by using green methods [19]. In particular, these environmentally friendly methods transform the bulk phase material into nanometer sizes. Top-down options are preferred in the industry because they are more straightforward, less expensive, and easy to scale up production with. Although it is a viable green technique and requires limited manual operation, particle functionalization will be more problematic, and the resulting nanoparticles, through these inexpensive routes, will be in a wide distribution of size and shapes, which directly limits their utilization for biological applications [9,20]. A wide range of nanoparticles have been synthesized by mechanochemical processing, which is an alternative technique to synthesize particles with a mean size as low as 4 nm, low agglomeration, narrow size distributions, and uniformity of crystal structure and morphology [21].

Conversely, bottom-up techniques (also known as wet chemistry routes) refer to the chemical synthesis of nanoparticles, wherein the primary nucleation of nanomaterials followed by growth and aggregation will finally result in desired nanoparticles with specific sizes and shapes [22]. Various materials, such as proteins, polysaccharides, and synthetic polymers, are used to prepare nanoparticles. The choice of matrix materials depends upon many factors including the required size of nanoparticles, surface characteristics, degree of biocompatibility, degree of biodegradability, and toxicity [2]. Moreno et.al reported the addition of acids in the sol-gel preparation of γ-Fe_2_O_3_/SiO_2_ to obtain single-phase γ-Fe_2_O_3_ magnetic nanoparticles with controllable particle size and size, while the distribution opens a new method for the design of nanocomposites for different applications [23]. The natural zeolite-type clinoptilolite NPs were synthesized by using the chemical solution method to investigate their degradation ability subjected to methyl orange [24]. Tsang et al. synthesized iron-based magnetic nanometer-sized particles (FeNi, Fe_3_C) using a wet chemical method (sequential spraying followed by chemical precipitation, and controlled pyrolysis), which is a promising nanocomposite catalyst for the production of fine chemicals in liquid-phase reactions [25]. Such types of magnetic nanoparticles are also crucial in several areas of the nanotechnology field. The simple thermal decomposition method was carried out to synthesize truncated cubic iron oxide nanoparticles [26] promising SPIONs for MRI contrast agents. Spherical dots of SPIONs, the size ranging from 7.8 to 17.9 nm, were synthesized via thermal decomposition methods [27]. Macromolecules, including block copolymers, surfactants, and ionic liquids are widely used as a template and structure-directing agent to synthesize magnetic nanoparticles [16,28]. Specially designed block copolymers are a boon for the controlled synthesis of nanoparticles. The hydrophilic section of the amphiphilic block copolymers interacts with inorganic precursors, and the hydrophobic block works as a template to synthesize the porous nanoparticles [29,30]. Highly biocompatible porous Fe_2_O_3_ microspheres have been synthesized with a double hydrophilic block copolymer, polyethylene-block-polyacrylic acid (PEO-*b*-PAA) with a high loading capacity of drug molecules, which could be promising nanoparticles for magnetically guided nanocarriers [31]. The hydrophilic and anionic PAA block strongly interacts with ferric ions. Nucleation of the Fe_2_O_3_ nanoparticles is expected in the presence of a precipitating agent in an aqueous solution. At the same time, the neutral PEO corona stabilizes the primary nanoparticles, which ultimately undergo self-assembly to form the microspheres of Fe_2_O_3_ at elevated temperatures. The various promising features of polymers have been reported, thus, can enhance the physical and chemical properties of materials for subsequent suitable potential applications [32].

In this study, the bottom-up approach was used to fabricate IONPs. The main advantages of this approach are producing a nanostructure with fewer defects, a more homogeneous chemical composition, a control reaction mechanism, size, and optimization. There are many reports on the synthesis and applications of iron oxides for wastewater treatment. However, the current work uses amphiphilic block copolymers to synthesize sub-50 nm iron oxide nanoparticles with pores. Unlike in the previous work, the F127 block copolymer plays a dual role as a structure-directing agent and a porogen. The pore size on the nanoparticles is almost similar to the size of polymeric micelles. The IONPs were characterized using different techniques, including Fourier transform infrared (FTIR) spectroscopy, scanning electron microscopy (SEM), transmission electron microscopy (TEM), Raman spectroscopy, x-ray photoelectron spectroscopy (XPS), x-ray diffraction (XRD) and cryogen-free physical property measurement system (PPMS). The nanoparticles possess an abundance of sites and surface area for reactions. The Rietveld analysis of x-ray diffractometer spectra shows the hexagonal phase symmetry of α-Fe_2_O_3_ nanoparticles. A thorough study was performed to explore whether the dyes degrade or adsorb the nanoparticles. The systematic study was performed on the adsorption of model dyes, Congo red (CR), using an UV visible spectrophotometer and LC-MS/MS triple quadrupole with an electrospray ionization mass spectrometer. It shows that a significant number of dyes adsorbed by the nanoparticles get degraded in the presence of the oxidizing agent, H_2_O_2_, one of the components of the Fenton reaction.

## 2. Results and Discussion

**Figure 1:**
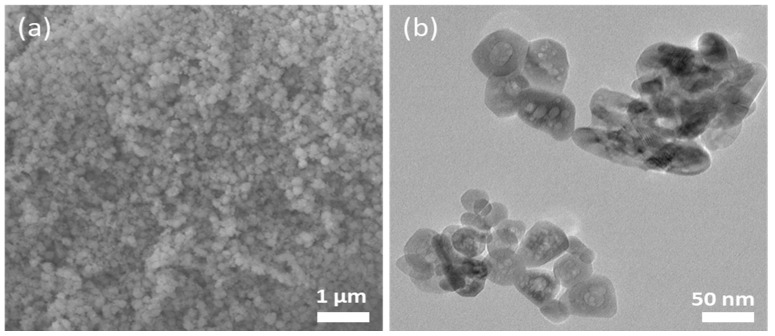
(**a**) SEM and (**b**) TEM images of the IONPs.

**Figure 2:**
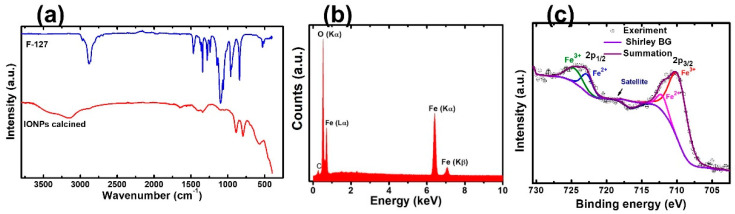
(**a**) FTIR spectra of polymer and IONPs. (**b**) EDX spectrum of IONPs. (**c**) XPS graph of Fe atoms showing the deconvolution at 2p_1/2_ and 2p_3/2_.

In nanoparticle synthesis, the surfactant undergoes self-assembled molecular clusters in a solution and is adsorbed by the interface between a solution and a different phase (solution/solids) [33]. We chose F127 as a template because of its availability, cheaper price, and known chemistry. It helps to control the size of the NPs, excels the adsorption efficiency, and enhances the superparamagnetic properties of magnetic nanoparticles [27,34]. The slow hydrolysis of urea releases ammonia, forming a complex with metal ions. The coordinated NH_3_ molecules interact with the polyethylene oxide chains (PEO) of the F127 units through hydrogen bonding, which is a driving force for the interaction of metal ions with the polymer [35]. Figure 1a shows the SEM images of IONPs. The particle size distribution graph of nanoparticles proves that F127 works as an effective structure-directing agent (Figure S1). The micelles of F127 wrap around the nanoparticles and prevent crystal growth. The aggregation of NPs is unavoidable in the absence of a polymer (Figure S2). TEM imaging was performed to explore the internal structure of nanoparticles. Multiple pores around 10 nm were in the TEM image (Figure 1b) showing the porogenic ability of F127. The TEM image shows a characteristic particle diameter of approximately 50 nm. As the concentration of the polymer in the solution is above the critical micelles concentration, then, colloidal nano aggregates (micelles) are formed. The inorganic precursors deposit around the micelles and the calcination remove the organic moieties leaving porosity on the inorganic nanostructure. The space occupied by micelles leads to the pores. The pore size is matched with the diameter of micelles of F127 in an aqueous solution [36]. The hydrodynamic diameter was found to be 310 nm with a polydispersity index of 0.3. The size is larger than the size obtained from electron microscopy. It is probably due to the aggregation of inorganic nanoparticles. Additionally, DLS measurements were carried out in solution, and the TEM measurements were made on a dry sample under a high vacuum. The nanoparticles have a −21 mV surface charge at pH 6.6. The removal of the polymer was monitored by FTIR spectra. The signature peaks of the polymer disappeared after calcination (Figure 2a). The characteristic peaks of FTIR at 573 cm^−1^, 787 cm^−1^, and 882 cm^−1^ are attributed to the Fe-O bond of Fe_2_O_3_. The EDX spectrum, shown in Figure 2b, confirms the presence of the O and Fe elements along with their respective characteristic X-ray emission lines (O: Kα 0.5249 keV, Fe: Lα 0.7048 keV, Fe: Kα 6.4006 keV, Fe: Kβ 7.0563 keV). Then, we carried out XPS measurements to further confirm the elemental composition and analyze the orbital state of the Fe element. Casa XPS v2.3 software was used to fit the XPS spectra. In this study, the binding energy of the Fe 2p_3/2_ and Fe2p_1/2_ are obtained at 710.67 eV and 723.89 eV, respectively, (Figure 2c), which are similar to previous reports [37,38,39]. The Fe 2p_3/2_ and Fe2p_1/2_ are further deconvoluted into Fe^2+^ and Fe^3+^ ions. The major peak at 710.67 eV confirms that the characteristic peak from Fe 2p_3/2_ core level electrons is attributed to Fe^3+^ [40] and the peak at 723.89 eV confirms that Fe^2+^ is in the octahedral sites. The Fe^2+^ ions are fundamental to determining the magnetic moment in the lattice [40,41]. The satellite peak associated with Fe 2p_3/2_, which appeared at approximately 719 eV, is evidence of the Fe^3+^ ions [38,40] supporting the Fe_2_O_3_ form of the IONPs. Corroborating these results with XRD and Raman analysis, confirmed that the prepared IONPs are dominant features of α-Fe_2_O_3_.

**Figure 3:**
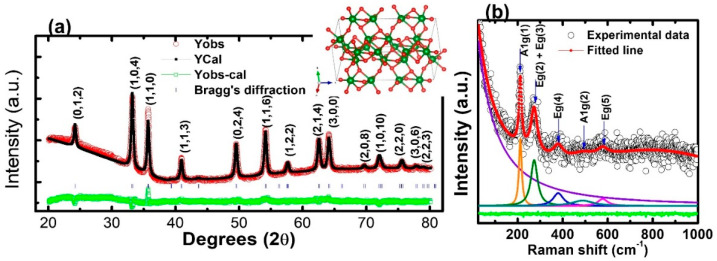
(**a**) Rietveld refinement of XRD spectra (inset: possible crystal structure of hexagonal IONPs). (**b**) Raman spectra of IONPs.

The XRD patterns with the Rietveld analysis (Figure 3a) indicate that the IONPs are polycrystalline and exhibit well-defined diffraction peaks matched with the JCPDS # 330664, which suggests a hexagonal (R3C) crystal structure. Various investigations have suggested hexagonal phase symmetry for the IONPs [42,43]. The crystallographic database file COD: 9016457 (R3C) CIF [44] was used for the reference phase of the sample, and the pseudo-Voigt function was used for the profile simulation in the FullProf suite software [45]. The Rietveld refinement analysis supported the hexagonal (R3C) phase symmetry of a sample, as reported for α-Fe_2_O_3_ [46,47]. The inset figure shows the crystal structure visualization of IONPs, showing red-colored O atoms and green-colored Fe atoms. However, both forms of the α-Fe_2_O_3_ and γ-Fe_2_O_3_ phases of the IONPs have also been reported [46,48]. The data of the refinement process such as lattice parameters (a & c), unit cell volume (v), angle α, β, γ, full-width–half maxima parameters (*viz*. U, V, W), R_wp_, R_exp,_ and χ^2^ are summarized in Table 1. Satisfactory results of χ^2^ = 3.69 were achieved, while the well-correlated parameters R_wp_ > R_exp_, the positive and negative values of u, v, w, and output plots imply the acceptance of our refinement process. The detailed satisfactory parameters for the Rietveld refinements are well explained by Brian [49]. The crystallite size (*D*) of the IONPs was estimated by Scherrer’s formula [50].

$$D=\frac{K\lambda }{\beta cos\theta }$$

where *K* is Scherrer constant (0.9), *λ* is the wavelength of the Cu *K*α radiation (1.5406 Å), *θ* is the diffraction angle, and *β* is the FWHM (full-width half at half maximum) of the corresponding peak. A value of *D* = 21.29 nm of the (104) peak was obtained. It is slightly smaller than the one for α-Fe_2_O_3_ previously reported by Tadic et al. [51], indicating that peak broadening which can be observed in the XRD peaks of the IONPs. The crystal size calculated from the XRD is less than the nanoparticle size estimated from the TEM images, which is expected in a polycrystalline material.

Figure 3b depicts the room temperature experimental Raman spectra (black hole) and fitted data (solid red line) for the IONPs. By analyzing the experimental data, using a Lorentzian function, five well-defined Raman modes were obtained in the wavenumber range 25–1000 cm^−1^. The deconvolution peaks suggest that the dominant features of the Raman shift result from a hexagonal α-Fe_2_O_3_. In the group theory to the point group of pure α-Fe_2_O_3_, seven vibrational modes are expected to be Raman active: 2A1g + 5Eg [52]. We observed approximate peaks of 213 cm^−1^ and 494 cm^−1^ associated with the A1g phonon modes, and three other peaks (~273 cm^−1^, 379 cm^−1^, 580 cm^−1^), which were related to the Eg phonon modes. The Raman active modes at room temperature for various forms of iron oxide are explained by Testa-Anta et al. [53]. These results corroborate the analysis of the X-ray diffraction peaks.

**Figure 4:**
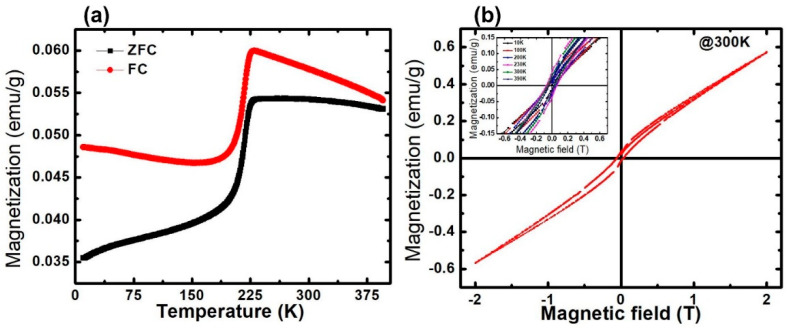
(**a**) Magnetization vs. temperature in FC and ZFC conditions recorded at 1000 Oe. (**b**) Magnetization vs. magnetic field hysteresis curve at 300 K (inset: magnetization vs. magnetic field at various temperatures (10–390 K) showing M_R_ and Hc).

Magnetic properties of the sample were measured at a wide range of temperatures (10–390 K) at 1000 Oe, under field-cooled (FC) and zero-field-cooled (ZFC) conditions, as shown in Figure 4a. Both the ZFC and FC curves increase with a decrease in temperature, though the rate of change in the magnetization is greater in FC, while after a certain temperature, the magnetization starts decreasing. For ZFC, when the temperature decreases, the magnetization increases slowly and reaches its maximum, which corresponds to the blocking temperature (T_B_), before decreasing sharply, which generates a large bifurcation at low temperatures. The magnetization of the FC curve achieves the maximum temperature of ~230 K, which is known as the saturation temperature (T*_sat_*). After the T*_sat_*, the spins gradually start freezing, showing spin glass-like behavior, which is attributed to the freezing of the disordered surface spins [26]. For both curves, we observed a decrease in magnetization below T_B_, confirming the SPIONs nature. The bifurcation is a common feature in the FC and ZFC curves in the frustrated magnetic systems, such as spin glasses and cluster glasses [54,55]. The divergence between ZFC and FC started at a temperature far above T_B_. This is due to the relatively broad size and shape distributions of the samples [56], which can be observed in the TEM micrographs. Further, the T_B_ values depend on the effective anisotropy and particle size, while T_B_ is the blocking temperature at which the thermal energy becomes compatible with the magnetic anisotropy energy barrier, where the particle goes in a superparamagnetic regime [57,58].

**Figure 5:**
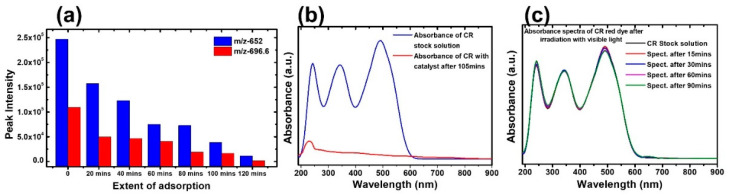
(**a**) Monitoring CR dye sodium salt and protonated structure in LC–MS. (**b**) Absorbance spectra of CR in the presence of IONPs. (**c**) Absorbance spectra in visible light in the absence of IONPs.

The magnetic hysteresis M(H) of the IONPs, measured up to ±2T at 300 K is shown in Figure 4b. The observed loop of M(H) indicates the superparamagnetic type of behavior that possesses a very low remnant and coercive field at the applied magnetic field (H). The Magnetic remnant polarization (M_R_) and the coercive field (H_C_) increase as the temperature increases, up to Tc, further increasing as the temperature starts to decrease. The sample shows a large M_R_ of approximately 0.0432 emu/g, with an H_C_ of approximately 0.0589T at 230 K. The observed values of M_R_ and H_C_ at various temperatures (10–390 K) is shown in the inset (Figure 5b) and summarized in Table 2.

IONPs are found to exhibit very strong adsorption of CR dye in the absence of light. Indeed, CR has a strong absorbance band at a wavelength of 486 nm. The absorbance intensity almost diminishes after 120 min (Figure 5b). The recent advances in the removal of dyes from wastewater and the possible adsorption mechanisms are explained in detail [59]. The stability of the CR solution in the absence of the catalyst was monitored both in the dark and following exposure to normal room light by measuring the absorbance every 15 min (Figure 5c). The absorbance remains unchanged, indicating that the dyes are very stable. To get more insight into the adsorption of the dyes of the Fe_2_O_3_ nanoparticles, a time-dependent LC–tandem MS/MS–MS equipped with a triple quadrupole mass analyzer and electrospray ionization (ESI) interface was used. The spectrum obtained at each time step of the adsorption process shows a reduction in the intensity of the 696.6 *m*/*z* peak, with a corresponding decrease in the intensity at the 652.7 *m*/*z* peak for the same step. This reduction in intensity was consistent for both masses of the CR dye molecule. The CR was confirmed based on the two masses observed at 696.6 *m*/*z* and 652.7 *m*/*z* for the CR dye sodium salt and protonated structures, respectively (Figure 5a). The sodium ions are either separated during the chromatography process or exchanged for protons during the electrospray process by the mass spectrometer. The semi-quantitative study was performed based on the molecular ion (696.6 Da) and the fully protonated CR molecule (652.7 Da). The mass spectra obtained at various stages after the treatment with Fe_2_O_3_ nanoparticles are shown in Figure S3. As the treatment of the solution with the adsorbent progressed with sampling at intervals of 20 min, the intensity of the peak reduced and became almost undetectable. This shows the strong adsorption ability of the nanoparticles toward dyes. Our materials show superior/comparable efficiency compared with previously reported works [60,61,62].

Fe_2_O_3_ nanoparticles are used to degrade organic pollutants in the presence of an oxidizing agent. In visible light, Fe_2_O_3_ nanoparticles get easily excited and generate electrons and holes. The photogenerated electrons reduce Fe^3+^ ions to Fe^2+^ ions. The Fe^2+^/H_2_O_2_ ions oxidize CR through the Fenton mechanism [63]. The CR was monitored after the addition of H_2_O_2_ in presence of visible light. The mass spectrum showed no peaks at *m*/*z*-696.6 and *m*/*z*-652.7, indicating complete degradation of the CR dye (Figure S4). The mass spectra of the CR stock solution and the sample solution treated with H_2_O_2_ show vast differences in their chemical compositions, as shown in Figure S4a,b). Multiple peaks were obtained in the degradation reaction as compared to the adsorption reaction. Peaks at *m*/*z* ratios of 305.2, 327.0, 349.1, 365.0, 371.3, 393.1, 415.1, 429.2, 608.3, 652.3, and 696.3 were not observed in the spectrum in the presence of H_2_O_2_ due to its complete degradation and the forming of other compounds of different masses to charge ratios. The catalytic degradation of CR and the subsequent degradation products have been well reported [64].

## 3. Experimental

### 3.1. Materials

Chemicals used in this experiment include urea (Sigma Aldrich, 99%) (St. Louis, MO, USA), block copolymer (Pluronic, F127; mol. wt. 12,500; Sigma Aldrich), iron nitrate hexahydrate (Fe(NO_3_)_2_·9H_2_O; Sigma Aldrich, ≥ 98%), hydrogen peroxide (H_2_O_2_; Alfa Aesar, 27% *w*/*w*) (Haverhill, MA, USA), Congo red (C.R.; Alfa Aesar, 99%), methanol, formic acid, and LC–MS grade water were purchased from Fisher Scientific (Fisher Scientific, Pittsburg, PA, USA).

### 3.2. Synthesis of IONPs

IONPs were synthesized using a polymer-controlled hydrothermal technique, and 0.30 g of the block copolymer was dissolved in 40 mL of deionized water using a magnetic stirrer (Wilmington, NC, USA). After the complete dissolution of the block copolymer, 1.25 g of Fe(NO_3_)·9H_2_O and 0.25 g of urea were added and stirred. The mixture was poured into an autoclave container (Dalian, China) and placed in a furnace at 90 °C for 12 h. The precipitation was centrifuged and washed four times using deionized water. Finally, the prepared sample was dried at 50 °C before calcination at 550 °C for 5 h at a ramping rate of 2 °C/min.

### 3.3. Characterization

Crystallinity and phase purity of IONPs were examined by X-ray diffraction (XRD) using a Rigaku Smart Lab X-ray Diffractometer (Rigaku Corporation, Japan), equipped with a Cu Kα radiation source (λ = 1.5406 Å), operating at an accelerating potential of 40 kV, and a tube current of 44 mA. The X-ray spectra were recorded in the range of a scattering angle (2θ) = 20 to 80 degrees, at a slow scan rate of 0.01. The sensitive and non-destructive technique of Raman spectroscopy was used to investigate the changes in lattice vibrations and the phase purity of the IONPs. The room temperature Raman spectra of the powder sample were collected via a Horiba spectrometer T64000 (Kyoto, Japan) with Ar-ion laser excitation (514.5 nm) and attached to an optical microscope with 80× resolution. Scanning electron microscopy (SEM) (JEOL, Tokyo, Japan) and an energy dispersive X-ray (EDX) (JEOL, Tokyo, Japan), excited by an electron beam of energy 20 kV in a high-resolution of 40K, were used. The internal structure of nanoparticles was observed using a JEOL TEM 1210 electron microscope (JEOL, Tokyo, Japan) at an accelerating voltage of 120 kV. An X-ray photoelectron spectroscopy (XPS) (Thermofisher Scientific, Waltham, MA, USA), from Physical Electronics 5600ci with ultra-high vacuum (~6 × 10^−9^ Torr), Al Kα source, analyzer diameter of 200 nm, and PHI Multipack 9.4, was used to study the chemical composition of the nanoparticles. The FTIR data were collected from the IRTracer-100, Shimadzu instrument in ATR mode (Kyoto, Japan). The field-dependent magnetic properties, M(H), and temperature-dependent magnetic measurements, M(T), of the IONPs, were recorded by the Quantum Design Dynacool fully automated, cryogen-free Physical Property Measurement System (PPMS) (Quantum Design, Inc., San Diego, CA, USA) within a temperature range from 10 K to 390 K, at a pressure < 10^−4^ Torr, and at the magnetic field range from 0 T to 2 T. The hydrodynamic diameter and zeta potential were monitored using a Zetasizer Nano MS (Malvern-UK) at 25 °C and in an aqueous solution.

### 3.4. Adsorption Test

A 20 ppm solution of the CR dye was prepared for the experiment at room temperature, using deionized water as solvent. The adsorption ability of the nanoparticles was investigated using 20 mL of the 20 ppm solution with 10 mg adsorbent in an enclosed 100 mL glass reaction vessel. At specific time intervals, 5 mL of the sample solution was withdrawn and centrifuged before measuring the absorbance readings. The effect of light and oxidizing agents on the adsorption/degradation of dyes was also studied for comparison. A total of 20 mL of the 20 ppm CR dye was treated with 10 mg Fe_2_O_3_ nanoparticles, using 0.5 mL H_2_O_2_ in the presence of light, with continuous stirring. The absorbance of CR at 486 nm was monitored using an UV-Vis spectrophotometer (Pasco Scientific SE-3607) (Pasco, Roseville, CA, USA). The in-depth study of the adsorption/degradation of dyes was performed by utilizing a liquid chromatography–tandem mass spectrometry (LC-MS/MS) system equipped with a triple quadrupole mass analyzer and an electrospray ionization (ESI) interface (Agilent, Santa Clara, CA, USA). The optimized experimental conditions for chromatographic measurement are shown in Table S1.

## 4. Conclusions

Polycrystalline IONPs were successfully synthesized with a hydrothermal technique using a block copolymer and urea under desired synthesis conditions. The size distribution of nanoparticles observed in electron microscopy images proved that F127 works as an effective structure-directing agent, while a characteristic particle diameter of ~50 nm was examined through transmission electron microscopy. The EDX spectrum confirmed the presence of O and Fe elements and the orbital states Fe^2+^ and Fe^3+^ ions were analyzed via the XPS spectra. The Rietveld analysis of the X-ray diffractometer spectra showed the hexagonal phase symmetry dominant features of α-Fe_2_O_3_, which is further supported by the Raman analysis. The magnetic property measurement (both M_H_ and M_T_) reveals the superparamagnetism on the IONPs with a maximum M_R_ and M_H_ of 0.0432 emu/g and 0.0589 T, respectively, at 230 K. The IONPs exhibited the very strong adsorption of the CR dye in the absence of light, the dye was almost diminished after 120 min. The systematic study of the adsorption of model dyes using LC–MS/MS triple quadrupole with electrospray ionization mass spectrometer showed a significant amount of dye was adsorbed on the nanoparticles and degraded in the presence of the oxidizing agent, H_2_O_2_. These properties of superparamagnetic behavior, with the strong ability to adsorb the dye, lay the foundations for designing and synthesizing magnetic oxide nanoparticles for wastewater treatment and other catalytic applications.

## Figures and Tables

**Table 1:** Refinement structural parameters and agreement factors for IONPs at room temperature with space group R3C.

Parameters	a (Å)	c (Å)	U	V	W	R_wp_ (%)	R_exp_ (%)	χ^2^	α = β	γ
Values	5.0302	13.7465	0.1806	−0.0475	0.1363	19.9	9.2	4.69	90	120

**Table 2:** M_R_ and H_C_ of the IONPs at various temperatures.

Temp. (K)	10	100	200	230	300	390
M_R_ (emu/g)	0.0196	0.0248	0.0338	0.0432	0.0357	0.0243
H_C_ (T)	0.0342	0.0247	0.0481	0.0589	0.0361	0.0208

## Data Availability

The data that support the findings of this study are available from the corresponding author upon reasonable request.

## Supplementary Materials

The following are available online at https://www.mdpi.com/article/10.3390/molecules28041914/s1, Table S1. Experimental parameters for LC-MS/MS measurement. Figure S1. Particle size distribution of nanoparticles from TEM images. Figure S2. SEM images of iron oxide nanoparticles synthesized in the absence of F127. Figure S3. The obtained mass spectra at various stages after the treatment with Fe_2_O_3_ nanoparticles. Figure S4. mass spectra of 20 ppm CR (a) before and (b) after treatment with IONPS and H_2_O_2_.

## Sample Availability

Samples are available from the authors.
