# Supplementary material for: Block Copolymer-Assisted Synthesis of Iron Oxide Nanoparticles for Effective Removal of Congo Red
Source: Molecules. 2023 Feb 17;28(4):1914. doi: 10.3390/molecules28041914 (PMC9964741; doi:10.3390/molecules28041914)
Supplement: Supplementary file 1 [file molecules-28-01914-s001.zip › molecules-2210806-supplementary.pdf]

molecules

Supporting Information

Table S1. Experimental parameters for LC-MS/MS measurement.

Parameter	*Experimental condition*
Column	HILIC (Hydrophobic Interaction Liquid Chromatography) Phenomenex Inc., Torrance, CA, USA
Column dimensions	150*4.6mm (3μm HILIC 200A0)
Mobile phase	Methanol/water (90:10) with 0.2% Formic Acid (F.A.)
Flow rate (mL/min)	0.5
Injection volume (μL)	50
Nebulizer pressure (psi)	15
Column temperature (°C)	25
Dry gas flow rate (L/min)	8
Gas temperature (°C)	300
Sheath gas temperature (°C)	250
Sheath gas flow (L/min)	11
Run time (min)	5

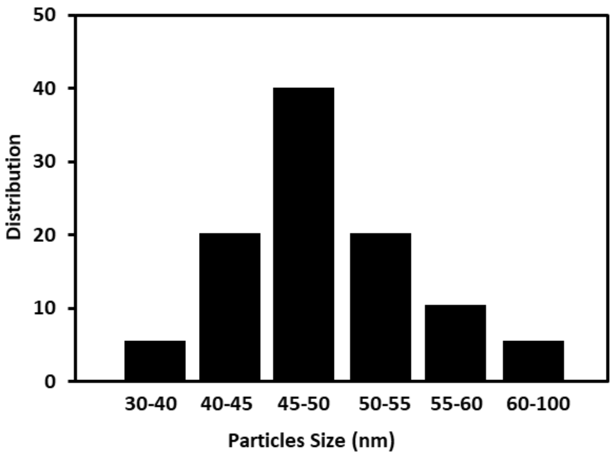

Figure S1. Particle size distribution of nanoparticles from TEM images.

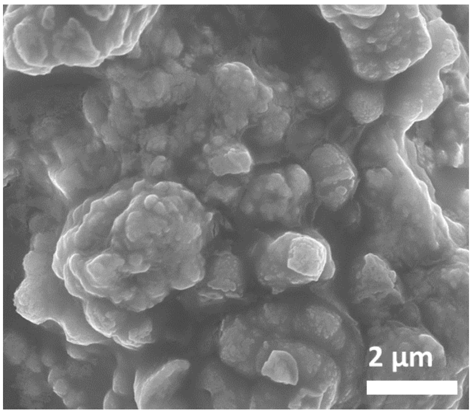

Figure S2. SEM images of iron oxide nanoparticles synthesized in the absence of F127.

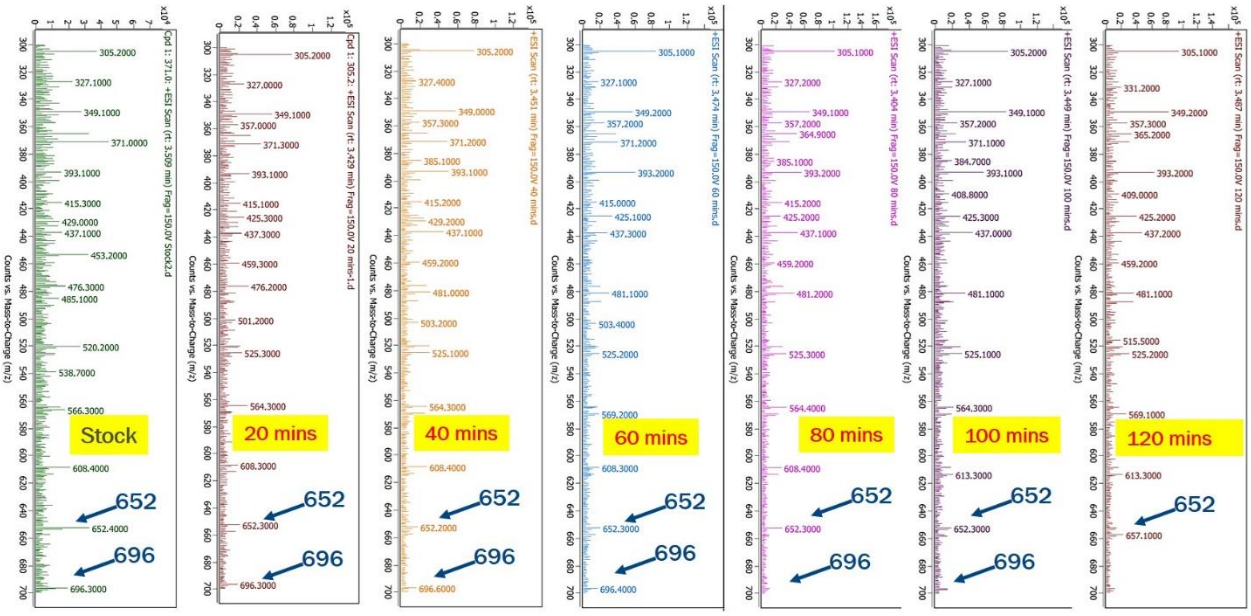

Figure S3. The mass spectra obtained at various stages after the treatment with Fe_2O_3 nanoparticles.

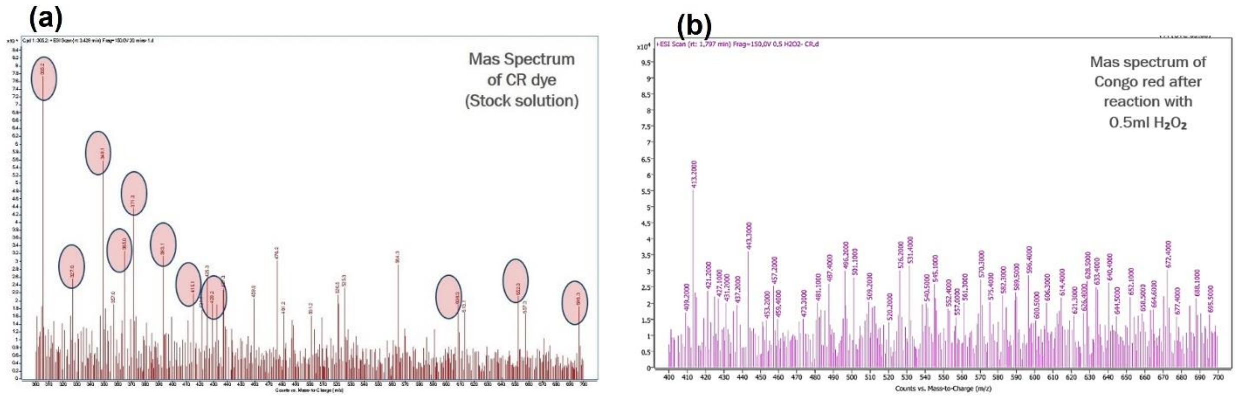

Figure S4. Mass spectra of 20 ppm CR (a) before and (b)after treatment with IONPS and H_2O_2.
